# Quantitative analysis of left atrial function in asymptomatic patients with b-thalassemia major using real-time three-dimensional echocardiography

**DOI:** 10.1186/1476-7120-9-38

**Published:** 2011-11-24

**Authors:** Constantina Aggeli, Ioannis Felekos, Emmanuel Poulidakis, Athanasios Aggelis, Dimitrios Tousoulis, Christodoulos Stefanadis

**Affiliations:** 1 Department of Cardiology, University of Athens Medical School, Hippokration Hospital, Athens, Greece

**Keywords:** Real-time 3D echocardiography, b-Thalassemia major, left atrial function

## Abstract

**Background:**

There is strong evidence that left atrial (LA) size is a prognostic marker in a variety of heart diseases. Recently, real-time three-dimensional echocardiography (RT3DE) has been reported as a useful tool for studying the phasic changes of the left atrial volumes. The aim of this study was to investigate the performance of the left atrium in beta-thalassemic patients with preserved left ventricular ejection fraction (EF) and no iron overload, using RT3DE.

**Methods:**

Twenty-eight asymptomatic b-thalassemic patients (32.2 ± 4.3 years old, 17 men) who were on iron chelating therapy, as well as 20 age- and sex-matched healthy controls underwent transthoracic RT3DE. The patient group had normal echocardiographic systolic and diastolic indices, while there was no myocardial iron disposition according to MRI. Apical full volume data sets were obtained and LA volumes were measured at 3 time points of the cardiac cycle: (1) maximum volume (LAmax) at end-systole, just before mitral valve opening; (2) minimum volume (LAmin) at end-diastole, just before mitral valve closure; and (3) volume before atrial active contraction (LApreA) obtained from the last frame before mitral valve reopening or at time of the P wave on the surface electrocardiogram. From the derived values, left atrial active and passive emptying volumes, as well as the respective emptying fractions were calculated.

**Results:**

Left ventricular EF (59.2 ± 2.5% patients vs. 60.1 ± 2.1% controls), E/A, E/E' were similar between the two groups. Differences in the LAmax, LAmin and LApreA between b-thalassemic patients and controls were non-significant, LAmax:(35.5 ± 13.4 vs 31.8 ± 9.8)cm^3^, LAmin:(16.0 ± 6.0 vs. 13.5 ±4.2)cm^3^, and LApreA:(25.4 ± 9.8 vs. 24.3 ± 7.2)cm^3^. However, left atrial active emptying fraction was reduced in the patient group as compared to the healthy population (34.3 ± 16.4% vs. 43.2 ± 11.4%, p < 0.05).

**Conclusion:**

RT3DE may be a novel technique for the evaluation of LA function in asymptomatic patients with b-Thalassemia Major. Among three-dimensional volumes and indices, left atrial active emptying fraction may be an early index of LA dysfunction in the specific patient population.

## Background

Cardiac involvement represents an important complication of b-thalassemia major, and results in increased mortality and morbidity rates. Disease mechanisms implicate iron infiltration of the heart structures due to frequent blood transfusions. Transfusion-dependent patients receive 20 times the normal intake of iron, which leads to iron accumulation and damage in the liver, heart, and endocrine organs [1]. Although iron chelating therapy has markedly improved outcomes, cardiac failure remains an important cause of death in thalassemic patients [2]. Therefore, early recognition of myocardial dysfunction is imperative, given the fact that majority of this patient group have preserved ejection fraction until late in disease process.

On the other hand, left atrial (LA) enlargement plays a pivotal role in the prognosis and management of various cardiovascular diseases, including coronary artery disease and heart failure [3,4]. Recently real-time three-dimensional echocardiography (RT3DE) has been integrated into clinical practice, providing unique data on phasic changes of LA volume during the cardiac cycle [5]. Furthermore, it has been validated against cardiac magnetic resonance (CMR) and has been proven as a reliable and robust method for the evaluation of LA volume; being more accurate than conventional 2D echocardiography [6].

The aim of the current study is to investigate the performance of the left atrium in b-thalassemic patients with preserved left ventricular ejection fraction (EF) and no myocardial iron overload employing RT3DE.

## Methods

### Study population

Twenty-eight consecutive asymptomatic patients (32.2 ± 4.3 yrs old, 17 men) with b-Thalassemia major were retrospectively studied, compared to twenty healthy age, sex- and BMI- matched controls. Thalassemic patients were on intense chelating therapy with deferipone (p.os) and desferioxamine (i.v/s.c). All patients were on sinus rhythm. Exclusion criteria included a medical history of smoking, arterial hypertension, diabetes mellitus, as well as the presence of heart failure and pulmonary hypertension symptoms (NYHA> Stage I). Structural and valvular heart diseases were also within the exclusion criteria. Additionally, patients with CMR-detected myocardial iron overload during the previous six-month scan were excluded from the study. A critical iron loading was defined as a T2* value less than the threshold of 20 ms (less than the lower limit value of the 95% confidence interval of the normal T2* value as described by Anderson et al. [7]), and values equal to or greater than this limit were considered to be uncritical. On the other hand, the control group was consisted of individuals with unremarkable clinical history and normal findings on clinical examination; none of them was receiving medication affecting the cardiovascular system. The study was approved by our institution ethical committee.

### Echocardiographic examination

Transthoracic echocardiographic studies, including, 2-dimensional echocardiography, pulsed Doppler, colour Doppler, tissue Doppler imaging, and RT3D echocardiography, were performed in all patients, after placing them in the left decubitus position. The echo study was performed 2-3 days after blood transfusion. For this purpose the Philips iE33 ultrasound machine was used which was fully equipped with a 2D transducer and a matrix array transducer for 3D data acquisition.

The 2D data were acquired using the 2.5 MHz S5-1 transducer. The dynamic range of this system was 40 Db. Conventional measurements were obtained from parasternal long-axis view. Peak velocities during rapid filling, atrial contraction (A), were measured using pulsed Doppler echocardiography from the apical 4-chamber view by positioning the sample volume at the tips of the mitral valve, and the E/A ratio was subsequently calculated. The early diastolic mitral annular velocity (E') was measured at the septal side of the mitral annulus using tissue Doppler imaging in the apical 4-chamber view. The ratio E/E' was estimated as a non-invasive LV filling pressure index.

### Real-Time Three Dimensional Echocardiography and Image analysis

**Figure 1:**
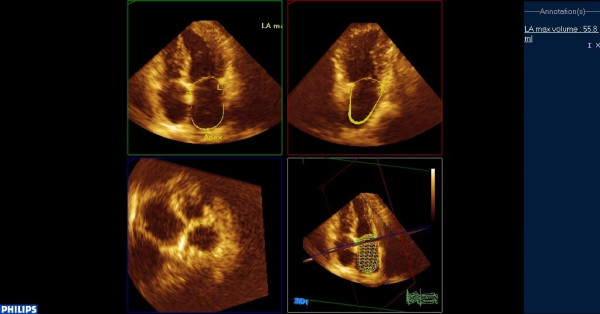
**Assessment of LA volumes using RT3DE**. Automatic border detection is obtained marking 5 reference points in the apical 2- and 4-chamber views (upper panel) and the LA 3-dimensional model is provided by the software (lower panel). The figure captures an end-systolic frame, from which left atrial maximum volume is derived.

**Figure 2:**
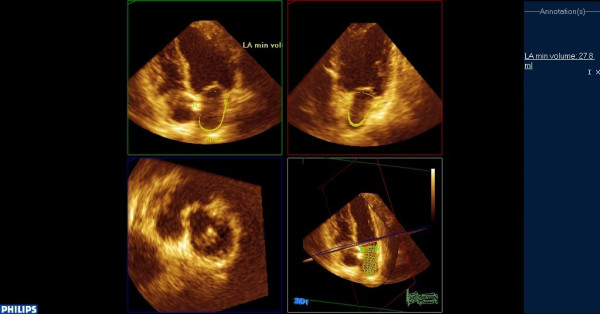
**Estimation of minimum left atrial volume in the same patient using RT3DE (end-diastolic frame)**.

**Figure 3:**
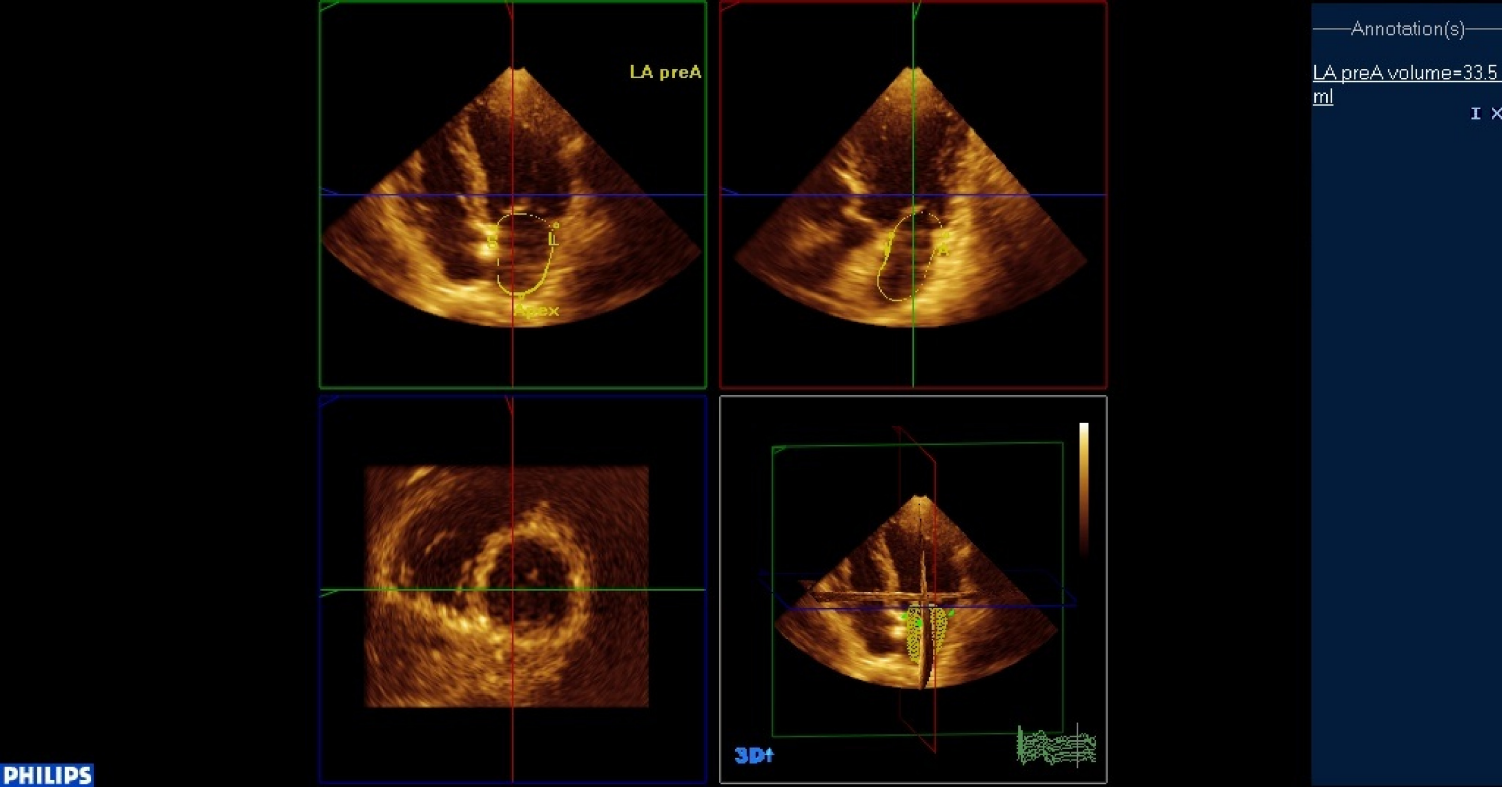
**Assessment of LA volume just before the atrial contraction (VpreA)**.

Real-time 3-dimensional echocardiography imaging was performed from the apical window using a commercial scanner equipped with a fully sampled matrix array transducer (x3-1), in the harmonic mode. Two full-volume data sets were acquired from an apical window over seven cardiac cycles, with a breath hold. The 3-dimensional data sets were transferred to a Q-Lab system (Philips Medical Systems, Andover, Massachusetts) for off-line analysis. Analysis of 3-dimensional images was based on a 2-dimensional approach, which relied on obtaining images from an apical 4-chamber view (Additional File 1). Subsequently a semi-automated tracing of the LA endocardial surface was generated in order to calculate the LA volume. Tracing was performed by marking five atrial points: the anterior, inferior, lateral, septal mitral annuli, and the midpoint of the LA posterior wall in a 4-chamber view. Once this was completed, the endocardial border was automatically delineated, and the LA volume was obtained throughout the heart cycle, resulting in LA volume-time curves (Figures 1,2,3). Manual modifications were made to correct the automatic tracings in some patients.

LA volumes were measured at 3 time points of the cardiac cycle: (1) maximum volume (Vmax) at end-systole, just before mitral valve opening; (2) minimum volume (Vmin) at end-diastole, just before mitral valve closure; and (3) volume before atrial active contraction (VpreA) obtained from the last frame before mitral valve reopening or at time of the P wave on the surface electrocardiogram.

All 3D LA volumes were independently assessed by two echocardiographers (C.A and I.F).

### LA volume definitions

LA passive emptying volume (PAEV) was defined as the maximum LA volume minus the LA volume before atrial contraction (PAEV = Vmax-VpreA). LA active emptying volume (AAEV) was defined as the LA volume before atrial contraction minus the minimum LA volume (AAEV = VpreA-Vmin). For the assessment of the LA function the following indicators derived from volumes were used: left atrium active emptying fraction defined as (AAEF) = (VpreA-Vmin)/VpreAx100; and LA passive emptying fraction (PAEF) = (Vmax-VpreA)/Vmax100.

### Statistical Analysis

Continuous variables are presented as mean ± standard deviation of the mean or as percentage where appropriate. Data were tested for normality by Kolmogorov-Smirnov test. The means of normally distributed data were compared using independent Student's t-test. A value of p < 0.05 was considered significant in all cases. All tests were two-tailed. Intra- and inter-observer agreement was assessed with the kappa statistic. Kappa statistic values are expressed as k ± std. error. Intervals of agreement are denoted as: kappa value greater than 0.800 denotes excellent agreement, 0.601-0.800 good agreement, 0.401-0.600 moderate agreement, 0.400 or less poor agreement. Data analysis was performed using the SPSS 17.0 statistical package for Windows (SPSS Inc, Chicago).

## Results

Twenty eight patients (32 yrs old, 17 men) as well as twenty healthy controls (31 ± 5.1 yrs old, 11 men) were enrolled in the study. All patients with beta- thalassemia were on chelating therapy and had no myocardial iron according to CMR. Patient demographics are illustrated in Table 1.

**Table 1:** Study population characteristics.

*Variable*	*Patients*	*Controls*	*P *
**Age (years old)**	32.2 ± 4.3	31 ± 5.1	0.4
**Sex (male/female)**	17/11	11/9	0.7
**BMI (kgr/m^2^)**	21.3 ± 3.8	22.1 ± 5.3	0.2
**BSA (m^2^)**	1.2 ± 0.3	1.3 ± 0.1	0.1
**Systolic BP (mmHg)**	112.3 ± 12.9	114.2 ± 12.8	0.6
**Diastolic BP (mmHg)**	72.3 ± 7.9	75.8 ± 6.1	0.1
**Heart Rate (bpm)**	77.2 ± 10.3	72.1 ± 8.2	0.07
**Medications**			
**Iron chelating therapy(n of pts)**	28	-	
**Biochemical Data**			
**Mean Serum feritin levels (μg/l)**	111 ± 84	-	
Signa-MRI T2*heart (ms)	36.9 ± 5.6	-	

Values are expressed as mean and standard deviation. In all case differences were not statistically significant (p = NS).

Measurements regarding conventional two-dimensional echo as well Doppler indices were similar between the 2 groups (tables 2, 3). The average LA diameter obtained by 2D-echo was 36.2 vs. 35.5 mm for the patient and control groups respectively. It should be noted that the participants had normal ejection fraction. There was no statistical difference between the two groups with regards to 2D measurements and Doppler findings (conventional and TDI derived).

**Table 2:** Echocardiographic data derived by conventional 2D measurements.

*Parameters *	*Patients*	*Controls*	*p *
**LA diameter (mm)**	36.2 ± 4.1	35.5 ± 3.5	0.5
**Ascending Aorta (mm)**	27.9 ± 2.7	29.3 ± 3.4	0.1
**End-diastolic diameter of the LV (mm)**	44.8 ± 3.4	46.1 ± 2.6	0.1
**End-systolic diameter of the LV(mm)**	29.4 ± 1.5	28,9 ± 1,2	0.4
**Ejection Fraction (%)**	59 ± 2.5	60.0 ± 2.1	0.1
**Intraventricular septum diameter (mm)**	8.2 ± 0.8	8.2 ± 0.9	0.9
**LV posterior wall diameter (mm)**	8.2 ± 0.8	8.2 ± 0.8	0.8
**RV diameter (mm)**	27.9 ± 1.9	27.4 ± 1.7	0.3

Values are expressed as mean and standard deviation. LA = left atrial; LV = left ventricle; RV = right ventricle. In all cases differences were not statistically significant (p = NS).

**Table 3:** Pulse Doppler and TDI parameters.

*Variable*	*Patients*	*Controls*	*p*
**E (cm/s)**	67.7 ± 5.9	70.45 ± 8.0	0.1
**A (cm/s)**	45.25 ± 5.9	45.6 ± 5.7	0.8
**E/A**	1.5 ± 0.1	1.6 ± 0.1	0.3
**E' (cm/s)**	13.9 ± 1.9	14.3 ± 2.0	0.5
**E/E'**	4.9 ± 0.8	4.9 ± 0.7	0.9
**IVRT (msec)**	59 ± 31	61 ± 28	0.6
**DT (msec)**	141 ± 38	136 ± 44	0.8

Values are expressed as mean and standard deviation. IVRT = isovolumic relaxation time; DT = deceleration time. In all cases differences were not statistical significant (p = NS).

**Figure 4:**
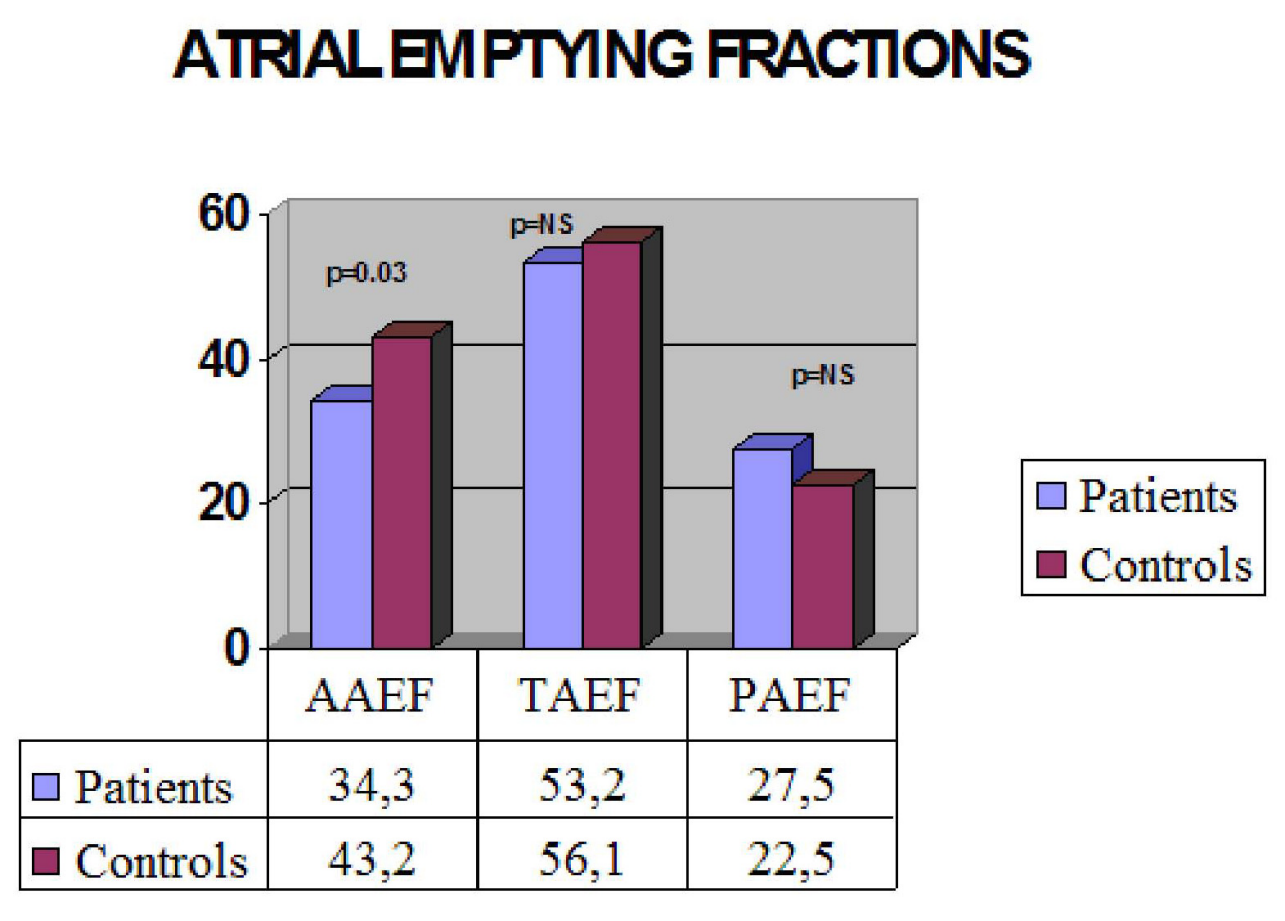
**Graph illustrating atrial emptying fractions (% mean values)**.

RT3DE reported no differences with regards to the phasic changes of atrial volumes (Table 4). The mean left atrial maximum volume assessed by 3D-echo was 35.5 vs. 31.8 ml, while the mean left atrial minimum volume obtained by 3D-echo was 16.0 vs. 13.5 ml, for the patient and control groups respectively. Moreover, mean left atrial emptying volumes both passive (10.3 vs. 7.5 ml for patients and control respectively) and active (9.3 vs. 10.8 ml for patients and controls respectively), were not statistically different. On the contrary, the mean atrial active emptying fraction was significantly lower in the patient vs. the control group (34.2 vs. 48.3 ml respectively), while the mean passive emptive fraction was similar for both groups (patients 27.5 vs. controls 22.5 ml), as illustrated in Figure 4.

**Table 4:** Left atrial volumes (ml) as calculated by RT3D

*P*	*Patients*	*Controls*
**Vmax**	35.5 ± 13.4	31.8 ± 9.8
0.2		
**Vmin**	16.0 ± 6.0	13.5 ± 4.2
0.1		
**VpreA**	25.4 ± 9.8	24.3 ± 7.2
0.7		
**AAEV**	9.3 ± 5.1	10.8 ± 4.7
0.3		
**TAEV**	19.7 ± 9.7	18.2 ± 8.3
0.5		
**PAEV**	10.3 ± 7.6	7.5 ± 3.7
0.1		

Values are expressed as mean and standard deviation. AAEV = active atrial emptying volume; TAEV = total atrial emptying volume; PAEV = passive atrial emptying volume; TAEV = total atrial emptying volume.

The agreement between the two readers was 0.918 ± 0.012 (0.894-0.942 95% C.I). Intra-observer agreement was 0.892 ± 0.017 (0.859-0.925, 95% C.I).

## Discussion

According to our knowledge, this is the first study that attempts to assess phasic changes of LA volumes in asymptomatic patients with b-thalassemia major by employing RT3D technology. As indicated by RT3D volumetric measurements, AAEF appears reduced in the patient population. This reflects abnormal systolic function of the LA, even in the absence of other affected echocardiographic parameters. This in turn could be explained by the fact that our patients were on intense chelating therapy, which maintained tissue iron levels within normal range (as illustrated by serum ferritin levels); therefore no iron accumulation was reported in the CMR evaluation. Indeed, studies have demonstrated that intense chelating therapy may prevent myocardial systolic function deterioration or even improve it [8]. Moreover, it should be noted that our patients did not have co-morbidities such as arterial hypertension or diabetes mellitus, which could potentially affect myocardial systolic and diastolic performance, and thus confound our results.

Iron toxicity is a major determinant of disease prognosis in b-thalassemic patients [9]. Although MRI is the gold standard in assessing early iron accumulation in the myocardium, echo is more widely available and cheaper. Monitoring ejection fraction can be useful, but its value is limited by the masking of ventricular dysfunction via the basal high cardiac output seen in chronic anaemia, and its late occurrence in the disease process [10]. Even modern contractile measures, such as tissue Doppler imaging, correlate poorly with cardiac iron. In a TDI study conducted by Silvilairat et *al*. [11], diastolic dysfunction was completely absent in patients who had ferritin levels below 2500 ng/ml. This observation was consistent with our results. Furthermore, data by Kremastinos [12] illustrated diastolic abnormalities only after the onset of cardiac failure. Margi et *al*. [13] have suggested strain indices as potential markers of early myocardial dysfunction in young asymptomatic patients, who nevertheless had myocardial iron as suggested by the reduced T2* values on MRI. Again in that study all conventional echo measurements failed to illustrate any significance between the patient and the healthy control groups.

On the other hand, left atrial volumetric assessment employing RT3D seems to offer an alternative approach as it can provide useful clinical information in early disease states, regardless of the left ventricular ejection fraction [5]. In our study, the impaired AAEF reflects early dysfunction of the left atrium, which could be attributed to the fact that the LA is formed by thin walls that are earlier affected as compared to the left ventricular wall. The early impairment of left atrial function in thalassemia major has also been implicated by studies in asymptomatic thalassemic individuals employing biochemical parameters such as atrial natriuretic peptides [14,15]. In addition to that, Trikas et *al*. have illustrated that LAEF was reduced in well-preserved thalassemic patients, a finding that was verified by our results. According to the same authors, this reduction was associated with reduced exercise capacity [16].

The echocardiographic evaluation of LA dimensions and performance is a matter of intense research. LA diameters as measured with 2D and M-Mode from the parasternal view tend to underestimate the true atrial dimensions, although they are useful and widely available [17]. Enlargement of the LA is often asymmetrical and may occur in the medial-lateral as well as the superior-inferior axes, due to limited anteroposterior axis enlargement by the thoracic cavity. These limitations led to the establishment of atrial volumes as more accurate indices for the evaluation of LA dimensions [18,19]. Although LA volumes are influenced by body type, this was not the case in our study since both groups had similar BMI and BSA. As suggested by Cameli [20] and other investigators [21], a relatively novel technique for the assessment of atrial function would be the utilization of strain rate imaging. As stated by the authors, this could provide a useful insight into the pathophysiology of various cardiovascular diseases, since it is a feasible and reproducible technique. It should be noted though that this method has major limitations, such as the inability of capturing a region of interest resembling the atrial shape or the confounding effects on the echo signal by structures surrounding the left atrium [20].

More recently, LA volume has been measured using real-time 3D echocardiography that has shown good agreement with other imaging modalities including magnetic resonance imaging [22,23]. Additionally, 3DE direct volumetric and 3D speckle-tracking methods give comparable and reproducible quantification of LV and LA volumes and function; this renders interchangeable application a viable option in daily clinical practice [24]. Left atrial volume assessment by 3D echocardiography has the most favourable test-retest variation with the least intra- and inter-observer variability compared with other echocardiographic techniques, with good correlations between biplane 2D and 3D measurements [25]. Moreover, RT3D can overcome limitations due to geometric assumptions, which is a main drawback of the two-dimensional approach [26]. For all these reasons, RT3D was chosen as the method of reference for the more accurate evaluation of LA volumes.

However, studies on 3D echo measurement of LA volume have been limited, and there is no consensus on the methods or comparisons with established normal values. In addition, the low frame-rates achieved by the X3-1 transducer constitute a major limitation. Furthermore, we did not employ strain and strain rate measurements, which could provide information on early atrial involvement in our study population [13,27]. Biochemical parameters such as natriuretic peptides have not been assessed, which could lead to correlations of clinical significance.

## Conclusions

Real-time 3D seems to be a novel method for the accurate assessment of LA phasic changes in asymptomatic patients with b-thalassemia major. Additionally, AAEF may represent an early index of impaired left atrial performance in the specific population. In turn, left atrial dysfunction could be related to the increased incidence of arrhythmias and reduced functional capacity during exercise in thalassemic patients. Our results imply that left atrial function should be thoroughly investigated by clinicians.

## Abbreviations

LA: left atrial; CMR: cardiac magnetic resonance; 2D: two-dimensional; RT3D: real-time three-dimensional; AAEV: active atrial emptying volume; PAEV: passive atrial emptying volume; AAEF: active atrial emptying fraction; PAEF: passive atrial emptying fraction.

## Competing interests

The authors declare that they have no competing interests.

## Authors' contributions in the manuscript

CA and IF in the data collection, CA in the laboratory analysis, EP and AA in statistical analysis, IF drafted the manuscript, CS provided critical review of the manuscript. All authors read and approved the final manuscript.

## Supplementary Material

Additional file 1 **3D full-volume dataset of a thalassemic patient, cropped to illustrate the 4-chamber view**. Click here for file

## References

[B1] Hershko C Iron loading and its clinical implications Am J Hematol 2007 82 12 Suppl 1147 8 [PMID: 17963253] 17963253 10.1002/ajh.21070

[B2] Hahalis G Alexopoulos D Kremastinos DT Zoumbos NC Heart failure in β-thalassemia syndromes: A decade of progress Am J Medicine 2005 118 957 967 10.1016/j.amjmed.2005.02.021 16164878

[B3] Douglas PS The left atrium: a biomarker of chronic diastolic dysfunction and cardiovascular disease risk J Am Coll Cardiol 2003 42 7 1206 7 10.1016/S0735-1097(03)00956-2 14522481

[B4] Suh IW Song JM Lee EY Kang SH Kim MJ Kim JJ Kang DH Song JK Left Atrial Volume Measured by Real-Time 3-Dimensional Echocardiography Predicts Clinical Outcomes in Patients with Severe Left Ventricular Dysfunction and in Sinus Rhythm J Am Soc Echocardiogr 2008 439 10.1016/j.echo.2007.09.002 17961977

[B5] Murata M Iwanaga S Tamura Y Kondo M Kouyama K Murata M Ogawa S A Real-Time Three-Dimensional Echocardiographic Quantitative Analysis of Left Atrial Function in Left Ventricular Diastolic Dysfunction Am J Cardiol 2008 102 1097 1102 10.1016/j.amjcard.2008.05.067 18929716

[B6] Artang R Migrino RQ Harmann L Bowers M Woods TD Left atrial volume measurement with automated border detection by 3-dimensional echocardiography: comparison with magnetic resonance imaging Cardiovascular Ultrasound 2009 7 16 10.1186/1476-7120-7-16 19335908 PMC2669050

[B7] Anderson LJ Holden S Davis B Prescott E Charrier CC Bunce NH Firmin DN Wonke B Porter J Walker JM Pennell DJ Cardiovascular T2 star (T2*) magnetic resonance for the early diagnosis of myocardial iron overload Eur Heart J 2001 22 2171 2179 10.1053/euhj.2001.2822 11913479

[B8] Farmaki K Tzoumari I Pappa C Oral chelators in transfusion-dependent thalassemia major patients may prevent or reverse iron overload complications Blood Cells Mol Dis 2011 47 1 33 40 Epub 2011 Apr 29 10.1016/j.bcmd.2011.03.007 21531154

[B9] Aessopos A Farmakis D Hatziliami A Fragodimitri C Karabatsos F Joussef J Mitilineou E Diamanti-Kandaraki E Meletis J Karagiorga M Cardiac status in well-treated patients with thalassemia major Eur J Haematol 2004 73 359 366 10.1111/j.1600-0609.2004.00304.x 15458515

[B10] Vogel M Anderson LJ Holden S Deanfield JE Pennel DJ Walker JM Tissue Doppler echocardiography in patients with beta thalassemia detects early myocardial dysfunction related to myocardial iron overload Eur Heart J 2003 24 113 9 12559943 10.1016/s0195-668x(02)00381-0

[B11] Silvilairat S Sittiwangkul R Pongprot Y Charoenkwan P Phornphutkul C Tissue Doppler echocardiography reliably reflects severity of iron overload in pediatric patients with beta thalassemia Eur J Echocardiogr 2008 9 3 368 72 17689292 10.1016/j.euje.2007.06.003

[B12] Kremastinos D Tsiapras DP Tsetsos GA Rentoukas EI Vretou HP Toutouzas PK Left ventricular diastolic Doppler characteristics in b-thalassemia major Circulation 1993 88 1127 1135 8353874 10.1161/01.cir.88.3.1127

[B13] Sciomer S Fedele F Gualdi G Casciani E Pugliese P Losardo A Ferrazza G Pasquazzi E Schifano E Mussino E Quaglione R Piccirillo G Early impairment of myocardial function in young patients with b-thalassemia major European Journal of Haematology10.1111/j.1600-0609.2008.01054.x 18284626

[B14] Derchi G Bellone P Forni GL Lupi G Jappelli S Randazzo M Zino V Vecchio C Cardiac involvement in thalassaemia major: altered atrial natriuretic peptide levels in asymptomatic patients Eur Heart J 1992 13 10 1368 72 1396810 10.1093/oxfordjournals.eurheartj.a060068

[B15] Brili SV Tzonou AI Castelanos SS Aggeli CJ Tentolouris CA Pitsavos CE Toutouzas PK The effect of iron overload in the hearts of patients with beta-thalassemia Clin Cardiol 1997 20 6 541 6 10.1002/clc.4960200607 9181265 PMC6655738

[B16] Trikas A Tentolouris K Katsimaklis G Antoniou J Stefanadis C Toutouzas P Exercise capacity in patients with beta-thalassemia major: relation to left ventricular and atrial size and function Am Heart J 1998 136 6 988 90 10.1016/S0002-8703(98)70154-1 9842011

[B17] Jiamsripong P Honda T Reuss C Hurst RT Chaliki HP Grill DE Three methods for evaluation of left atrial volume European Journal of Echocardiography 2008 9 351 355 17658300 10.1016/j.euje.2007.05.004

[B18] Lang RM Bierig M Devereux RB Flachskampf FA Foster E Pellikka PA Recommendations for Chamber Quantification: A Report from the American Society of Echocardiography's Guidelines and Standards Committee and the Chamber Quantification Writing Group, Developed in Conjunction with the European Association of Echocardiography, a Branch of the European Society of Cardiology J Am Soc Echocardiogr 2005 18 1440 1463 10.1016/j.echo.2005.10.005 16376782

[B19] Jenkins C Bricknell K Marwick TH Use of Real-time Three-dimensional Echocardiography to Measure Left Atrial Volume: Comparison with Other Echocardiographic Techniques J Am Soc Echocardiogr 2005 18 991 997 10.1016/j.echo.2005.03.027 16153532

[B20] Cameli M Caputo M Mondillo S Ballo P Palmerini E Lisi M Marino E Galderisi M Feasibility and reference values of left atrial longitudinal strain imaging by two-dimensional speckle tracking Cardiovascular Ultrasound 2009 7 6 10.1186/1476-7120-7-6 19200402 PMC2652427

[B21] Vianna-Pinton R Moreno CA Baxter CM Lee KS Tsang TS Appleton CP Two-dimensional speckle-tracking echocardiography of the left atrium: feasibility and regional contraction and relaxation differences in normal subjects J Am Soc Echocardiogr 2009 22 3 299 305 10.1016/j.echo.2008.12.017 19258177

[B22] Keller AM Gopal AS King DL Left and right atrial volume by freehand three-dimensional echocardiography: in vivo validation using magnetic resonance imaging Eur J Echocardiogr 2000 1 55 65 10.1053/euje.2000.0010 12086217

[B23] Tops LF van der Wall EE Schalij MJ Bax JJ Multi-modality imaging to assess left atrial size, anatomy and Function Heart 2007 93 1461 1470 10.1136/hrt.2007.116467 17934005 PMC2016891

[B24] Kleijn SA Aly MA Terwee CB van Rossum AC Kamp O Comparison Between Direct Volumetric and Speckle Tracking Methodologies for Left Ventricular and Left Atrial Chamber Quantification by Three-Dimensional Echocardiography Am J Cardiol 2011 10.1016/j.amjcard.2011.05.042 21784385

[B25] Maddukuri PV Vieira M DeCastro S Maron MS Kuvin JT Patel AR Pandian NG What Is the Best Approach for the Assessment of Left Atrial Size? Comparison of Various Unidimensional and Two-dimensional Parameters with Three-dimensional Echocardiographically Determined Left Atrial Volume J Am Soc Echocardiogr 2006 19 1026 1032 10.1016/j.echo.2006.03.011 16880098

[B26] Jenkins C Chan J Hanekom L Marwick TH Accuracy and feasibility of online 3-dimensional echocardiography for measurement of left ventricular parameters J Am Soc Echocardiogr 2006 19 1119 28 10.1016/j.echo.2006.04.002 16950466

[B27] Hamdy AM Use of strain and tissue velocity imaging for early detection of regional myocardial dysfunction in patients with beta thalassemia Eur J Echocardiography 2007 8 102e109 10.1016/j.euje.2006.02.004 16564231

